# Making sense of low-complexity domains: The 2025 Lasker Basic Science Award

**DOI:** 10.1073/pnas.2519778122

**Published:** 2025-09-11

**Authors:** William G. Kaelin

**Affiliations:** ^a^Sidney Farber Professor of Medicine, Department of Medical Oncology, Dana-Farber Cancer Institute and Brigham and Women’s Hospital, Harvard Medical School, Boston, MA 02116; ^b^HHMI, Chevy Chase, MD 20815

## Abstract

Up to 10 to 20% of the proteome contains regions with much lower amino acid diversity than would be expected by chance. This year’s Lasker Basic Science Award is given to Steven McKnight and Dirk Görlich for their pioneering work on such low-complexity domains (LCDs). They showed, using a variety of elegant approaches, that such LCDs can form homotypic and heterotypic interactions that lead to reversible phase separations in cells. These phase separations, which in the laboratory manifest as hydrogels and in cells as membrane free structures (liquid condensates) such as P bodies and stress granules, form hubs for molecular processes such as transcription and messenger RNA (mRNA) splicing and also underlie the selectivity of nuclear pore complexes (NPCs), which act a barriers to large molecules unless they are escorted by specific LCD containing nuclear transporters that interact with LCD containing nucleoporins within NPC channels. Naturally occurring LCD mutations linked to neurodegeneration and other diseases cause the formation of irreversible (rather than reversible) LCD polymers, resulting in insoluble, amyloid-like, fibrils, underscoring the critical importance of these domains.

High school students are taught that the primary sequence of proteins determines their folding and hence function. Curiously, however, upward of 20% of the proteome consists of polypeptides that contain a very limited repertoire of amino acids and that are thought to be structureless (1). Steven McKnight from UT Southwestern and Dirk Gorlich at the Max Planck Institute are being awarded the Albert Lasker Basic Science Prize for their discoveries related to the biochemistry, structure, and function of low-complexity (LC) domains in proteins.

I will begin with the contributions of Steven McKnight. McKnight has repeatedly, throughout his career, conducted experiments that are highly original and creative. A chemical screen conducted at McKnight’s institution, University of Texas Southwestern, identified a sulfonyl-hydrazone, 5-aryl-isoxazole-3-carboxyamide (which I will refer to as “isox” for short) that could promote embryonic stem cell differentiation (2). To get at the underlying mechanism, in 2012 he made a biotinylated version of isox (b-isox) and did pulldown experiments of mammalian cell extracts using streptavidin agarose (3). To his surprise, b-isox captured a large but specific set of cellular proteins, even in the absence of streptavidin agarose. He quickly realized that this was because b-isox rapidly came out of solution and precipitated under the conditions of his pulldown assays. Mass spectrometry analysis identified over 100 isox-binding proteins, including many that are known to associate with various intracellular granules, including many RNA-binding proteins that contain LC domains and associate with RNA granules (3). He went on to show that in all cases examined it was the LC domain that was necessary and sufficient for capture by b-isox. Since the solubility of b-isox was both concentration and temperature-dependent, he made the leap to ask whether LC domains might mediate phase transitions in proteins. Remarkably, he discovered that fusing various LC domains to fluorescent proteins enabled those fusions to form semisolid hydrogels in vitro and that a hydrogel formed by a given LC domain could homotypically or heterotypically trap other LC fusion proteins and that the heterotypic trapping was LC domain specific (3).

Many of the proteins captured by b-isox contained multiple copies of a [G/S]-Y-[G/S] tripeptide (3). Using site-directed mutagenesis he confirmed that a threshold number of these repeats was needed for biogel formation. More importantly, he used transmission EM and X-ray diffraction to show that the hydrogels consisted of amyloid-like fibers resulting from cross-beta structures (3). He discovered, however, that the LC fibers, unlike amyloid fibers, were readily reversible without the need for detergents, as though poised to interconvert between soluble and insoluble states. Finally, he showed that b-isox crystals fortuitously form surface ridges with the proper spacing to specifically capture cross-beta structures.

In 2013 McKnight showed that unphosphorylated RNA polymerase II, and not phosphorylated RNA polymerase II, could be captured by b-isox, and confirmed that the unphosphorylated RNA poly II C-terminal domain (CTD) could form heterotypic interactions with the LC domains of “unstructured” transcriptional activation domains and that such heterotypic interactions could be disrupted by CTD phosphorylation in vitro (4). He used mutagenesis of a model transactivation domain to show that its ability to form a hydrogel was tightly correlated with its ability to bind to the CTD and to activate transcription. He also did studies in support of the idea that local recruitment of LC-containing DNA-binding and RNA-binding to proteins to DNA and RNA, respectively, would increase their local concentration and favor biogel formation.

In 2015 McKnight devised a clever chemical footprinting method based on SILAC (Stable Isotope Labeling with Amino Acids in Cell Culture) mass spectrometry with or without chemical acetylation of surface accessible amino acids to show that the cross-beta fiber formed by a prototypical LC protein were the same in vivo and in vitro (5). In 2017 McKnight, working with Robert Tycko, used nuclear magnetic resonance (NMR) to solve the structure of a prototypical LC domain from the fused in sarcoma (FUS) RNA-binding protein at greater resolution (6). It showed that the LC cross beta structure, in contrast to classic amyloid fibers, was likely reversible because it lacked core hydrophobic interactions found in the latter. Moreover, McKnight did proof of concept experiments showing again that the formation of liquid-like droplets and hydrogels by the FUS LC domain could be regulated by phosphorylation.

In 2021 McKnight extended his findings to the many intermediate filaments (IFs) that contain LC domains in their so-called head domains (7). He found that a mild detergent known to dissolve condensates and biogels also disassembled IFs. Using NMR, coupled with intein chemistry and segmental isotope labeling, he showed that these LC head domains also form cross beta structures and hydrogels. Notably, he showed that certain naturally occurring autosomal-dominant intermediate filament mutations linked to diseases such as Charcot–Marie–Tooth disease caused the inappropriate stabilization of these structures, causing them to be more “amyloid-like” (7). The idea of a disease-causing mutation of an LC domain causing disease by their inappropriate aggregation was first suggested by experiments reported in 2009 by James Shorter (8) and in 2015 by Anthony Hyman and Simon Albert (9).

To test the predictions from his structural insights, McKnight in 2022 used intein chemistry and peptides with artificial amino acids to decrease the number of potential main chain hydrogen bonds available for cross beta sheet interactions (10). Decreasing the number of hydrogen bonds decreased gel formation. Conversely, he showed that many LC mutations linked to diseases such as Charcot–Marie–Tooth, Frontotemporal Dementia, and Paget’s Disease resulted in the elimination of a proline, the one amino acid that cannot form main chain hydrogen bonds. He showed that loss of the proline, as his model predicted, promoted gel formation, which could be rescued by eliminating neighboring main chain hydrogen bonds.

I’ll now move to Dirk Gorlich. Prior to the work of Dirk Gorlich, several groups had shown that certain nuclear transporters interact with low-complexity FG-rich domains within the nucleoporins that make up the nuclear pore complex (11–17). In 2001 Gorlich meticulously measured nuclear pore passage rates for nuclear transport receptors compared to other “normal” proteins of similar size and shape (e.g., green fluorescent protein) (18). He noticed that the rate for the transporters was so high that it was close to free diffusion through the central channel, while the much slower passage of normal proteins could only be explained by the presence of a permeability barrier. He proposed that this barrier is formed by the low-complexity FG repeat domains of the nucleoporins that multivalently interact with each other, forming a transport-selective phase that is a good solvent for the transporters (because they bind FG motifs) but a bad solvent for normal macromolecules. Pore-passage in this model reflected partitioning into the barrier and exit on the trans-side. This model would also explain why cargoes of very different sizes can be transported and that the barrier remains intact even when large objects pass.

In 2002 Gorlich reported that the set of cellular proteins that bound to a hydrophobic phenyl Sepharose column were remarkably similar to the collection of nuclear transporters captured on a Ran-GTP column (19). He performed in vitro nuclear import assays that indirectly supported the idea that the hydrophobic channel of a nuclear pore was both essential for preventing passive diffusion, insofar as it was disrupted by a mild detergent, and also essential for facilitated transport initiated by nuclear transport receptors, which interact with the FG repeats as stated above.

In 2006 Gorlich reported that the FG repeat domain of the yeast nucleoporin Nsp1p could form hydrogels and, through mutagenesis studies, that the FG motifs not only bound nuclear transport receptors but also mediate cohesive interactions that are necessary for hydrogel formation and yeast survival (20). He later confirmed by nuclear pore reconstitution experiments that the permeability barrier indeed relies on cohesive FG repeat interactions (21). In 2007, he reported that Nsp1p hydrogels could serve as a stringent diffusion barrier for large proteins (22). Remarkably, however, nuclear transport receptors entered the gel with kinetics similar to the passage times measured in intact cell nuclei. More importantly, a nuclear transporter enhanced by three orders of magnitude the barrier-entry of an artificial cargo protein fused to a cognate import signal. He reasoned that FG motif-binding by the rather hydrophobic transport protein transiently disrupts hydrophobic FG–FG barrier interactions as the transporter enters and traverses the channel. Release of cargo on one side of the pore or the other would, based on earlier work by Gorlich and others, then be coupled to a RanGTPase cycle.

In 2021 Gorlich succeeded in creating an artificial nuclear pore barrier consisting of 52 repeats of a 12 mer GLFG peptide (23). En route, he discovered that complete omission of proline from the repeat spacers resulted in the formation of irreversible amyloid-like fibers, in keeping with analogous findings from McKnight. The hydrogel formed by this synthetic GLFG repeat peptide served as a barrier against passive transport, but enabled facilitated transport by nuclear transporters, including import of Ran-GDP by NTF2, import of a suitably tagged cargo by Importin beta, and export of a suitably targeted cargo by Exportin I. Remarkably, the engineered permeability barrier also allowed for RanGTP-dependent exit of an importin cargo and the RanBP1 and RanGAP-dependent exit of an exportin cargo.

Scientists, like laypeople, tend to ignore or dismiss things that are initially hard to understand and are prone to using disparaging adjectives to describe them, such as noise, random, and junk. Although “low complexity” is literally correct insofar as it refers to the amino acid variation of low-complexity domains (LCDs), the elegant studies of McKnight and Gorlich illustrate that it is perhaps a bit of a misnomer in terms of the biological complexity and physiological importance of these common, yet enigmatic, domains. Their work helps us understand, biochemically, biophysically, and structurally, how these LCDs give rise, under physiological conditions, to phase separations and liquid condensates (e.g., P bodies and stress granules), and under pathophysiological conditions, to insoluble fibers and plaques (Fig. 1). These phase separations form hubs for certain types of chemical reactions, such as RNA splicing and transcription, and channels for intercompartmental protein transport, such as nuclear and peroxisomal import (24). Their work also helps us to understand how, in some cases, even a single amino acid change in a low-complexity domain can lead to disastrous outcomes, such as neurodegeneration.

**Fig. 1.**
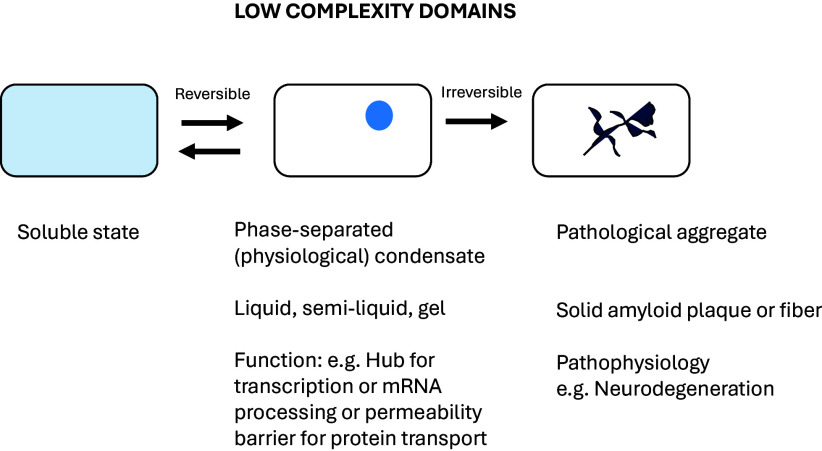
Model for different states assumed by LCDs and biological implications.

In recent years there has been a bit of a pendulum shift from mechanistic reductionist biology to descriptive systems biology, driven in part by powerful technologies that predictably generate data (although not necessarily knowledge) when applied to the right kinds of samples. Similarly, there has been a shift from biochemistry to molecular biology and genomics. The work of McKnight and Gorlich is a testament to curiosity-driven research, in their cases carried out by resourceful investigators who fearlessly and creatively blended biochemistry (with a capital “B”), biophysics, genetics, cell biology, and structural studies to arrive at their answers. Reading their papers I was constantly reminded of the geneticist Seymour Benzer, who was fond of saying that “a good experiment should be pretty and witty” (25). I think Benzer would applaud this year’s Lasker awardees.

## Data Availability

There are no data underlying this work.
